# On the Successful Employment of Belladonna as a Prophylactic during the Prevalence of Epidemic Scarlatina

**Published:** 1844-01

**Authors:** 

**Affiliations:** Valenciennes


*On the successful employment of Belladonna as a prophylactic during the prevalence of Epidemic Scarlatina.* By M. Stievenart, of Valenciennes, (*Bulletin de l'Academie Royale de Medicine*, February, 1843.)—An epidemic scarlatina ravaged, during the winter of 1840–1, several villages in the neighborhood of Valenciennes, when Dr. Stievenart was induced to try the prophylactic properties which belladonna is said to possess against this disease. In a small village, out of 250 individuals, 200 took belladonna, and were all preserved from the attacks of scarlet fever. Of the 50 others, 14 were siezed with the disease, and four of them died. At the village of Curgies, Dr. Stievenart administered the belladonna to the children at the public school, and allowed them to continue at their lessons and have communication with the other children of the village. All to whom the belladonna was administered escaped the scarlet fever; but a few who refused to take it were seized with the disease.

The belladonna was administered in two forms; in solution, or as a powder. Two grains of the recent alcoholic extract of belladonna were dissolved in an ounce of any aromatic infusion, and of this two drops were given to a child of one year old daily for nine or ten days. An additional drop was given for every additional year of age. The largest daily dose was, however, limited to twelve drops. When the belladonna was given in the form of a powder, half a grain of the powder of the root was mixed with a small quantity of sugar, and divided into ten doses. One of these was given morning and evening, to children of from one to two years old; two powders, morning and evening, to those from three to five; three powders to those from six to nine; four to those from ten to fourteen; and five to adults.

These small doses never produced the toxicological effects of belladonna; in fact, they scarcely ever had any marked action on the animal economy. In five or six cases Dr. Stievenart observed a rash, similar to that of measles; and, in a few other cases, headache with dilatation of the pupils, dryness of the throat, and slight sore throat, but which had no resemblance to that of *scarlatina anginosa.* In all the others, no sensible or apparent effect resulted from the administration of the remedy.

Dr. Stievenart generally continued the use of the remedy for from nine to ten days; in some cases, it was given for fifteen days. He thinks this period sufficiently long to put the system under the influence of the preservative powers of the remedy; but recommends to return to it if the epidemic return or break out again with renewed violence.

A few of the recorded instances in which belladonna has been successfully administered as a prophylaxis, are noticed. Bayle, in 1830, published a notice on this subject, by which it appears, that of 2027 individuals to whom belladonna was administered, 1948 were preserved from scarlet fever, and 79 were attacked with it. Dusterberg, by means of belladonna administered for two weeks, preserved from scarlet fever all those who took the medicine. In order to ascertain the real value of the remedy, he purposely omitted to administer it to one child in every family, and this child alone, according to his report, was seized with the disease. He adds, however, that scarlet fever occasionally siezed a child who had only been taking the remedy for three or four days, but was always in this case mild, and often only manifested its presence by desquamation ensuing. Reuch, physician of the Military Hospital for Children in the Tyrol, after eighty-four children were siezed with scarlet fever, was induced to try the prophylactic effects of belladonna on the remaining sixty-one children. With a single exception, all were preserved from its attacks, though the disease was raging around. Schenk, Berndt, Kohler, Meglin, De Lens, and other distinguished physicians, speak in equally high terms of the prophylactic properties of belladonna. *Edinburgh Med. and Surg. Journal*, October, 1843.

